# Predictions, Uncertainty, and Collective Epistemic Work: How Projected Futures Informed and Misinformed Enactments of Covid-19

**DOI:** 10.1177/03063127251351336

**Published:** 2025-07-11

**Authors:** Tobias Olofsson

**Affiliations:** Lund University, Lund, Sweden

**Keywords:** predictions, Covid-19, epistemic object, epistemic work, pandemic, herd immunity

## Abstract

By connecting an uncertain present to a potential future, predictions and other forms of projected futures construct meaningful contexts on which actors can lean when seeking to act in the face of uncertainty. This article outlines the background and careers of many, and often contradicting, futures that informed the collective work to define and represent Covid-19 in Sweden during the first half of 2020. Through an analysis of press briefing transcripts, in-depth interviews with centrally placed informants, and a timeline of Covid-19–related events, debates, and policies in Sweden, this article outlines how enactments of Covid-19 evolved over time—from straightforward comparisons to past experiences, to repurposed models intended to make the pandemic calculable, to survey-based extrapolations produced by the Public Health Agency. The article demonstrates how a combination of contextual factors and a continuously evolving knowledge base led some enactments to become more influential than others, allowing them to influence evolving decisions and strategies. The article highlights the role of competing voices and perspectives in the collective epistemic work performed during the pandemic and explores Covid-19 as a multiple entity composed of a patchwork of data and assumptions. Depending on what futures informed them, these enactments varied from catastrophic and dystopian, to hopeful promises of an eventual return to normality.

Reporter: Yesterday your colleague was asked about the figures that came from Oxford that showed that Sweden had the highest number of diseased per capita in the previous week. Your colleague hadn’t seen the numbers, have you? What is your view on them?

Tegnell: Yes! We have discussed what these numbers really mean. These are people who have died, they’re not just numbers. And you have to relate them to a whole lot of things. If you take the numbers of diseased per day, in Sweden these numbers vary vastly between days, it moves between 4 and 140 cases, so it is not a relevant figure. Countries are simply in different stages [of the pandemic]. You can see this in the serological surveys. Finland has a certain percentage, Spain another. So, you must be careful with what you cannot know. They are not proof of who is doing a better or worse job. Such conclusions will have to wait. (Press briefing May 20, 2020)

This exchange between a reporter and Swedish state epidemiologist Anders Tegnell—held during one of the daily press briefings organized by the Swedish Public Health Agency during the spring and early summer of 2020—is indicative of how numbers, trajectories, and uncertainty contributed to the enactment of Covid-19 (Mol, 2003; see also Law, 2004) in public discourses and debates. During the spring of 2020, Sweden, like many other countries, was in a first wave of Covid-19 infections, and the question of what could and could not be known about the new disease was at the forefront of epistemic work at the time. The uncertainties brought by the new virus (SARS-CoV-2) and the disease it caused (Covid-19) made their enactments fraught with challenges. Questions such as how Covid-19 spread, how severe it was, and how one might best mitigate and manage its consequences were intensely debated by experts, journalists, and decision-makers that sought to navigate an ocean of test results, reports, research preprints, surveys, and models.

In this article, I highlight the collective nature of the epistemic work produced during the pandemic, as well as the interactions between different views and enactments of Covid-19. In doing so, I pay particular attention to how the future shaped the enactments of Covid-19 produced by experts, government agencies, reporters, and others during the first half of 2020, when information was especially scarce and unreliable. The futures outlined in predictive modelling, scenarios, and other forms of projections played a central role in how decision makers approached the pandemic and often shaped the ways in which SARS-CoV-2 and Covid-19 were represented—not only as a quantified ‘datademic’ (Didier, 2025), but also as competing projected futures with bearing on public discourses and policymaking (Brooks-Pollock et al., 2021; Engelmann et al., 2023; Rhodes & Lancaster, 2022). As the above exchange between the reporter and Tegnell demonstrates, ideas about how present events relate to potential future outcomes, and vice versa, were also at the center of the public discourse and the competing enactments—and potential enactments—that sought to define Covid-19 in the collective understanding of the pandemic and its consequences.

By focusing on the role of future-informed enactments in the collective work to define what was, at the time, an elusive and uncertain epistemic object (Knorr-Cetina, 2001), namely Covid-19, this article explores how the pandemic’s present became known through its relation to a projected pandemic future. As I discuss below, projected futures reflect on the present by linking the here-and-now to a realm of potential future presents (Luhmann, 1976; see also Bell & Mau, 1971). By studying the contents and careers of influential and salient projections, this inquiry offers a novel perspective on the collective epistemic work—albeit unequally shared—through which the pandemic was made known and actionable. Drawing on insights from medical STS as well as sociological and anthropological studies of futures, the article approaches the Covid-19 pandemic as a multiple object composed of many, and often contradicting, enactments. Recognizing the multiplicity of enactments—each informed by particular present futures—opens the door to asking which ones among many were able to influence public discourse and strategy during the pandemic. For example, what information informed the Swedish Public Health Agency’s position that Covid-19 would best be addressed through existing pandemic preparedness plans (see, e.g. Public Health Agency, 2019; see also Anderberg, 2021) and WHO frameworks (see, e.g. World Health Organization, 2017) and how did their information differ from that used to justify lockdown policies elsewhere?

Moreover, by focusing on the interplay between epistemic work, the enactments produced therein, and the futures informing this process, one can move beyond the tendency to reduce the events of the pandemic to a matter of alleged incompetence or questionable political and epistemological commitments. Such accounts are relatively common in existing scholarship on the pandemic and pandemic policies. This includes studies proposing a connection between Boris Johnson’s hypermasculinity and the UK’s pandemic policies (Waylen, 2021) as well as studies arguing that Swedish decision makers rejected evidence that went against their favored laissez faire approach to pandemic management (Perlstein & Verboord, 2021; Svensson & Rodriguez, 2021) and that the ‘epistemic monopoly’ exerted by the Swedish Public Health Agency gave selfishly motivated technocrats an outsized influence over the policies introduced against the pandemic (Bylund & Packard, 2021). In these accounts, individual decision-makers become the endpoints in stories of why the pandemic unfolded the way it did. Consequently, the pandemic described in these accounts becomes an epidemio-political drama centered around the psychological constitution of a few decision-makers, while the inquiry into the pandemic is reduced to a question of whether their decisions were right or wrong. This focus on individual decision-makers, however, underestimates the crucial role played by the many different voices present in the cultural and socio-material settings in which the pandemic unfolded. In contrast, this article focuses on the epistemic work and the enactments of the pandemic that informed not only decision-making and strategizing but also public discourse and understanding at the time. And it does so with particular attention to the case of Sweden where the reliance on a ‘soft lockdown’ (Giritli Nygren et al., 2020) and voluntary social distancing—in contrast to the curfews, school closures, and similar policies introduced elsewhere—positioned the public as an integral and active part of the effort to understand and adapt to the pandemic.

## The future in the present: Expectations, narratives, and numbers

By depicting a potential future, projections give meaning to the present as they connect events in the here-and-now to their potential outcomes. As Bell and Mau (1971, p. 18) remark, ‘images of the future are of critical importance in influencing which of the alternative futures becomes present reality’. This relation between present and future is especially evident in times of crisis, when actors need to find ways to foretell what will come in order to adapt and seek to apprehend catastrophe before it materializes. However, the future is an important social domain also in calmer times. Any future-oriented act must therefore negotiate the tension between acting towards the future and the uncertainty of outcomes, a tension that Braun (2015) calls the ‘futurity paradox’.

While this paradox has been central to much scholarship on how the future is produced and mobilized in economic sociology (see, e.g. Beckert & Richard, 2018) it has received far less attention in STS. Instead, STS researchers tend to focus on how broad and widely held narratives about the future shape actors’ expectations and engagements with science and technology in the present. Jasanoff’s (2015, p. 6) widely cited writing on the future as sociotechnical imaginaries—‘collectively held and performed visions of desirable futures’ that shape national and social identities around science and technology—has been highly influential in steering STS scholarship in this direction (for related but alternative approaches see Borup et al., 2006; Pollock & Williams, 2010; Tutton, 2021). This abstracted and broad view is relatively silent regarding what is unfolding on the ground and between actors when new projections of the future are created and communicated. This is, instead, a central theme in how economic sociologists and economic anthropologists approach the role of futures in shaping the present (see e.g. Beckert & Richard, 2018; Ferry & Limbert, 2008). Scholars in these traditions describe the work behind producing, using, and communicating projected futures—work that Fine (2010) refers to as ‘futurework’. Influential studies of such work include research on financial forecasts and the ways in which they allow financial actors to construct ‘fictional expectations’ that act as ‘as-if-statements’, by standing in for actual knowledge of the future (Beckert, 2013; see also Leins, 2018). Other prominent studies in this tradition include research on financial discounting (Doganova, 2018), cost-benefit-analysis (Espeland, 1998; Porter, 1996), and risk management protocols and credit rating technologies (Carruthers, 2013; Rona-Tas, 2017). In some domains, futures take the form of eschatological narratives describing inevitable change (Geiger, 2020), in others they are the products of standardized tools, such as risk management protocols (Power, 2007) that allow actors to reconstruct the future as something more ‘structured and manageable than the uncertain world modern society has to face’ (Esposito, 2015, p. 97). In addition, economic sociologists and historians have highlighted that projected futures can provide their creators with the means to claim roles within those futures (Andersson, 2018).

Central to this scholarship are studies that explore how projections enable opportunities for actors to enact the future as a chain of events that together constitute a coherent whole. These enactments—for example, of markets as constituted by strings of past, present, and future prices—can be used by actors to negotiate uncertainty and to mobilize resources or political support for their version of the future. This is evident when proponents of a new mine, for example, use financial forecasts and environmental impact assessments to win stakeholders’ support (Olofsson, 2020) or when emergent industries use projections of future success to justify decisions and actions in the present (Reinertsen & Asdal, 2019). However, although such enactments allow actors to act as if the future was knowable, they nevertheless remain uncertain (Carruthers, 2013) and may even become blind alleys. The 2007–2008 financial crisis is a well-documented example of this.

As recent scholarship has shown, the lead-up to the 2007–2008 financial crisis involved financial analysts, investors, and risk assessors relying on quantitative risk assessment tools that performed the future in ways that allowed them to enact financial markets as calculable, albeit risky, phenomena (Carruthers & Kim, 2011; Lockwood, 2015; MacKenzie, 2011). However, as Katzenstein and Nelson (2013) point out, these risk management tools often conflated calculable risk and incalculable uncertainty in ways that significantly overestimated the predictability of the market. This is especially relevant, economic sociologists and historians argue, in the case of statistical models, which are expected to depict scientifically justified and unambiguous definitions of situations (Svetlova, 2012, p. 157) and are often granted additional confidence based on a wide-spread trust in numbers as mechanically objective (Porter, 1996; see also Reinertsen & Asdal, 2019).

### Epidemiology futures

Using tools such as predictive modeling and scenarios, epidemiologists produce images of epidemics and pandemics as trajectories outlined in tables, graphs, and maps describing the geographical and temporal distribution of infections (Anderson, 2019; F. Lee, 2020; Rhodes & Lancaster, 2022). Examples of how projected futures have been used to estimate the course and consequences of pandemics can be found across the board of recent pandemic events, including the 2008 H5N1 (avian) influenza outbreak, the 2009 H1N1 (swine) influenza pandemic (Forster, 2012), the 2013–2016 Ebola outbreak in West Africa (Parker et al., 2019), and the 2015–2016 Zika virus epidemic (F. Lee, 2020). In each case, projections of the epidemics’ and pandemics’ futures informed policies and interventions. However, the use and contents of these projections were not without controversies. The models and algorithms used to describe and predict the spread of Zika virus, for example, have been shown to neglect to account for data scarcities, uncertainties, and indeterminacies affecting the predictions (Lee, 2023). And in the case of the H1N1, the WHO’s overestimation of the severity of the pandemic and the resulting stockpiling of antiviral pharmaceuticals led critics to question whether the organization had faked its projections to favor industry profits (Abeysinghe, 2014). As I will discuss in detail below, the futures described in projections do not always align and may even come into conflict with alternative projections or with other forms of enactment (see Leach & Scoones, 2013).

The 2001 foot-and-mouth disease outbreak among livestock in the UK offers an illustrative example of when diverging futures clash during an epidemiological crisis. Writing on the epistemological conflicts between modelers and experts during the crisis, Law (2008) describes how a team of modelers were able to persuade the Blair government that the veterinary experts working to manage the outbreak had underestimated the situation and were not in control of it. According to the modelers, the disease’s rate of transmission (R_o_) was much higher than veterinarians had estimated. Based on this, they forecasted that the epidemic would be far more dire than the veterinarians had predicted. Supported by their projections, the modelers convinced the government to override the advice of the national veterinary officer and introduce an unprecedented policy to cull all animals on all farms contiguous to those with confirmed outbreaks. This policy was later called into question and the modelers criticized for relying on improbable biological assumptions and inaccurate population data in their models (Kitching et al., 2006). Some have gone as far as calling the events a ‘salutary warning of how models can be abused in the interest of scientific opportunism’ (Mansley et al., 2011, p. 493). However, as scholars interrogating the use of models and scenarios during the Covid-19 pandemic have pointed out, the power of models to override other forms of representation or enactments is not merely a matter of abuse and opportunism. Rather, it is a consequence of how graphs and numbers—expressing doubling times and the numbers of confirmed and predicted cases—portray pandemics as compelling, depoliticized, and urgent stories. Stripped of assumptions, contextual messiness, and uncertainties, these representations stress the need for a swift action in the face of an envisioned catastrophic future (Anderson, 2021; Lee et al., 2024; Rhodes & Lancaster, 2022). Nevertheless, while examples like these highlight the uncertainties and complexities hiding beneath the surface of projected epidemiological futures, projections remain important points of orientation that state actors, organizations, and individuals can refer to when seeking to navigate epidemiological threats and crises.

Below, I explore the trajectories of projections that became influential or salient in the public debates and discussions of the pandemic in Sweden. I outline where the projections came from, what futures they described, and how they were received, debated, and assessed by public health officials, experts, and journalists at the time. In doing so, I highlight the role that the future played in public attempts to navigate the uncertainties surrounding the pandemic’s past, present, and future, and I ask which projected futures were able to influence Swedish pandemic strategy and which futures were rejected.

## Exploring pandemic futures: Case and methods

As I have noted above, the Swedish response to Covid-19 relied primarily on existing pandemic preparedness plans focusd on mitigating the effects of a pandemic outbreak. The responsibility for implementing the mitigation plan resided with a broad array of independent national, regional, and municipal agencies. The National Board of Health and Welfare, for example, was responsible for providing standards on the treatment and mitigation of Covid-19 in healthcare and care settings, and for coordinating resources among the regional healthcare administrations and municipal level care providers. The Public Health Agency, in turn, issued general recommendations and drafted policies. The government, in keeping with Swedish administrative tradition (see Ahlbäck & Wockelberg, 2015) abstained from intervening in the agencies’ work, but coordinated the efforts, assigned tasks to the agencies, and drafted policies to address the consequences of the pandemic beyond the realm of public health, including economic, financial, and labor market policies (see Olofsson & Vilhelmsson, 2022).

While several agencies were involved in the work to mitigate the pandemic in Sweden, the Public Health Agency together with the National Board of Health and Welfare, and the Civil Contingencies Agency played prominent roles through their constant presence in the press briefings organized by the Public Health Agency.^1^ These press briefings, which began on March 4 and continued throughout the pandemic, were a central arena for debates about Covid-19, SARS-CoV-2, and the recommendations introduced to mitigate them. Because of this, the press briefings were immensely important for the collective understanding of the pandemic. During the briefings, the agencies provided updates on the latest epidemiological trends—such as the number of new cases, the number of patients receiving intensive care, and the current and expected demand on healthcare resources—and provided updates on policies and recommendations. In addition to this, spokespersons for the three agencies also responded to questions posed by journalists. According to the Civil Contingencies Agency (2020) up to 1 million viewers and 1.5 million listeners tuned in to the daily press briefings in the spring of 2020. Because of their continuity throughout the pandemic and their large audience (Sweden has approximately 10 million residents), the press briefings offer an important window into how portrayals of Covid-19 and its past, present, and future evolved over time, with new epidemiological data, preliminary research results, or salient controversies.

To understand both the work behind and the context beyond the press briefings, this article combines an analysis of press briefing transcripts with five in-depth interviews conducted with centrally placed informants within and outside the Public Health Agency, alongside a timeline of Covid-19-related events, debates, and policies in Sweden from the earliest days of the pandemic to the lifting of the last pandemic policies on May 5, 2023. Together, these materials open a window into the ways in which projected futures contributed to the pandemic policies in Sweden at the time.

Transcripts of the press briefings were obtained through a freedom of information request made to the Public Health Agency. The interviews were conducted in the first half of 2021 and include two interviews with senior level Agency officers, one interview with an epidemiologist working with scenarios and predictions on the regional level, and two interviews with scholars and researchers who made prominent contributions to the discussions and debates on Covid-19. The interviews and press briefing transcripts were analyzed using the qualitative software program NVivo in two waves of coding. The first wave consisted of descriptive line-by-line coding (Strauss & Corbin, 1998) and the second wave consisted of the thematic arrangement of the codes from the first wave into code groups describing themes related to how the pandemic was enacted, the scenarios and predictions that supported these enactments, discussions and critique of scenarios and predictions, and moments in which scenarios and predictions were discussed in relation to knowledge claims and strategies. Excerpts from interviews and press briefings are presented in the coming sections based on how well they summarize and illustrate the themes and events described.

## Predictions and collective epistemic work before and during the first wave of Covid-19 in Sweden

During the first wave of infections in 2020, a broad array of futures, including model-based predictions, scenarios and other forms of projection, guided actors across society, experts, decision-makers, reporters, and the public in their struggle to manage the disease and the threats it posed. Some of these futures were based on comparisons to past experiences, others relied on models developed for other diseases that were repurposed and tweaked so that they could be used to make the limited data available for Covid-19 projectable, and a third group of futures were based on extrapolations from survey data. In this section, I explore examples of these three modes of projection (comparison, repurposing, and extrapolation) and consider how they contributed, first, to the portrayal of the pandemic and, second, to the conflicts that unfolded over which images best matched the actual trajectory of Covid-19 and what this meant for the interventions introduced against the disease. The section ends with an account of a conflict over the use of Covid-19 mortality predictions in enactments of and the criticism leveraged against it by some experts and practitioners.

### Comparisons: Early enactments and Covid-19 as a low-risk disease

When reports of a pneumonia outbreak of unknown origin in Wuhan, China started circulating in late December 2019, few expected what was to come. For example, while the WHO first picked up on local reports about the virus on December 31, it did not declare it an international public health emergency until January 30 (see Olofsson & Vilhelmsson, 2022). Likewise, in Sweden, it took several weeks before the Public Health Agency announced that the novel virus posed a public health risk. And when the Agency issued its first update about the virus on January 16, it came with a prediction saying that the risk of the disease, spreading from China to Sweden, was very low (Public Health Agency, 2020). When interviewed, a senior officer with the Agency explained the reasoning behind this prediction, saying that the initial reports coming out of China were not ‘all that worrying’ and did not raise many eyebrows:

You have to remember that new cases of rare viruses from China is not news. That’s something that tends to happen every year. Sometimes several times a year. It’s usually different types of influenza viruses, but you also get other viruses turning up there or in other parts of Asia. And I’d say that in that context, the very first reports weren’t all that worrying. (Interview D)

There was, in other words, no indication at the time that SARS-CoV-2 would be any different from other viruses discovered in the region, and this expectation informed how the virus was portrayed and enacted at the time. As state epidemiologist Anders Tegnell noted in his memoirs, an outbreak of pneumonia of unknown origin had been discovered and successfully suppressed in Ulanqab in Inner Mongolia as recently as November 2019, and only a few months prior, a case of H5N6 (a strain of avian influenza) had been discovered in Beijing (Tegnell & Härgestam, 2023, p. 13). When viewed through the lens of past experiences of seemingly comparable viruses, there were no signs that this new disease would be any different.

Likewise, when it was reported that the new virus belonged to the Corona family, experts still leaned on past comparable examples. For example, when comparing Covid-19 to other viruses in the same family, Agency officers and other experts only made minor revisions to their initial projections. Tegnell explains in his memoirs (Tegnell & Härgestam, 2023), for example, that his first reaction upon learning that the new disease was caused by a Coronavirus was to think that the virus would probably follow the same course as SARS and MERS—two coronaviruses that had caused limited outbreaks in 2002–2003 and 2012. Tegnell was not alone in drawing parallels to these previous outbreaks. As one virologist explained:

My initial thought was that, alright, this might turn out to be like SARS, with outbreaks primarily in hospitals and care facilities at the same time as we’ll be able to contain it with relative efficient measures. (Interview A)

However, over time signs started emerging that gave experts reason to reconsider their expectations. For the virologist quoted just above, this happened when reports surfaced that the outbreak in Wuhan was not limited to hospitals, but that the disease was spreading more broadly within the city. This, the virologist reasoned, made the comparisons to SARS and MERS less feasible. As more data became available from Wuhan and as new outbreaks became known—including the outbreaks onboard the cruise ship Diamond Princess and in Lombardy, Italy—these doubts were confirmed, and it soon became clear that Covid-19 was not going to be a repetition of the SARS and MERS outbreaks.

### Repurposing: Models, uncertainty, and lockdown

As it became clear that Covid-19 would not be as easily contained as SARS or MERS had been, experts scrambled to find better ways of enacting the disease; an important part of this work was to find ways to use the limited information available about what was happening, ‘on the ground’ and use it to predict what was to come. Instead of the projections resting on an assumption that the new disease would follow the familiar path of other viruses, these new efforts left comparison aside and focused on repurposing existing models and using them to overcome the uncertainties around Covid-19. As the disease had no previous history in humans, projecting its spread and consequences required that modelers first filled in the gaps and unknowns, either by finding alternative data that could stand in for actual observations or by making assumptions about how the disease would spread, at what rate, and with which consequences. As a result, many enactments of Covid-19 at this time were informed by predictions that relied on models that combined the little data available with assumptions informed by what was known about other viruses, such as influenza. A well-known example of this form of projection, which uses repurposed influenza-based models, is the set of predictions outlined in a report published by the Imperial College London’s Covid-19 Response Team on March 16.

At the heart of this report was an individual-based simulation model that had originally developed to support pandemic influenza planning. To adapt the model for Covid-19, the team combined assumptions drawn from previous influenza epidemics with the limited data available from China and from people returning from China on repatriation flights (Ferguson et al., 2020). The respecified model was then used to predict the spread, intensive care demand, and mortality from Covid-19 in the UK and US under different policy scenarios, ranging from no interventions at all to a broad combination of non-pharmaceutical interventions. According to the report, the only viable alternative for the UK was to implement a combination of case isolation policies, society-wide social distancing, and either household quarantine or school and university closures. Less invasive alternatives, such as maintaining the so-called mitigation strategy in place at the time, was predicted to lead the intensive care demand to soar to eight times more than the available supply and result in a minimum of 250,000 deaths (Ferguson et al., 2020, p. 16).

Almost a month later, on April 15, a team of Swedish scholars published an article preprint in which they made similar predictions for Sweden. The team had adopted the Imperial College team’s individual-based model, fitted it with Swedish population data and used it to predict that Covid-19 would cause 96,000 deaths by July 1, 2020, unless Sweden quickly implemented a nationwide lockdown, in which case the mortality would drop to 40,000 in the same period (Gardner et al., 2020). Like the Imperial College team’s report, this preprint portrayed Covid-19 as a catastrophe-in-the-making which could only be avoided through ‘prompt and decisive action’. The authors argued: ‘Whereas the predictive value of our model should become much clearer over the next two weeks, suppressive measures should be taken more immediately to have a substantial effect’ (Gardner et al., 2020, p. 18). However, while these and similar reports and projections contributed to the Johnson government’s decision to introduce a lockdown (Boin et al., 2021; Evans, 2022), Swedish policymakers did not heed the call.

Part of the reason Sweden maintained its softer approach was that the Public Health Agency had found the individual-based models used by the Imperial College team and the Swedish team to be too uncertain (Olofsson et al., 2022). As a result, they chose instead to abide by existing pandemic plans. In his memoirs, state epidemiologist Anders Tegnell recalls the reasons why the Agency doubted the predictions made by Ferguson and colleagues: ‘at the time the report was published, we knew very little about Covid-19, which, in my assessment, made the values of the different variables [in the model] uncertain’ (Tegnell & Härgestam, 2023, p. 164). However, uncertainty was not the only reason why the Agency doubted the prediction. As one officer explained, the model and the way it was specified was also questioned:

I think we, like many others, realized early on that there were a lot of assumptions [in the models] and that the background to these assumptions was very loose. It felt like they, when deciding what parameters to use for different variables, had opted for the more extreme ones, so to say, and because of this they had produced quite extreme results overall. (Interview E)

The Agency’s doubts meant that they ignored calls for prompt and decisive actions. However, the question of whose vision on the pandemic’s past, present, and future was the most correct would remain a salient and contentious topic in the Swedish debate. To the modelers, according to whom Covid-19 would soon overburden the healthcare sector and cause death and suffering not seen in Sweden since the 1918 pandemic, the gap between what they saw unfolding in their models and the information that the Agency was communicating through its policies and recommendations would only lead to disaster. In the UK, similar tensions between what the models were predicting and the policies in place led many modelers to turn to activism in an attempt to influence decision-makers to take stronger action (Rhodes & Lancaster, 2022). A similar shift from predicting to acting took place in Sweden as a fierce debate started over what Covid-19 was, what its consequences would be, and which policies should be used against it. Among those calling for an end to Sweden’s soft approach and for the introduction of a lockdown were a group of scholars, authors, and medical doctors whom the media named ‘The 22 researchers’. In an open letter published on April 14 in Sweden’s largest daily newspaper *Dagens Nyheter*, the group offered a scathing review of the Public Health Agency’s work and called upon the government to step in and retake the reins from the Agency’s civil servants, whom they deemed lacked ‘any talent for predicting or suppressing the epidemic’ (Carlsson et al., 2020). On the other side of the debate was the Agency and its supporters, who were acting based on their own forecasts of the pandemic.

### Extrapolation: The Public Health Agency’s forecasts

The Agency’s questioning attitude toward predictive modeling, STS scholars have argued (Lee et al., 2024), reflects a broader division between two epistemic cultures in epidemiology: mathematical epidemiology and social epidemiology. While modelers tend to belong to the former culture, many of the senior officers at the Agency ascribe to the latter and to the associated prioritization of hands-on and contextual approaches over theoretical models. This includes Tegnell, whose past experiences include creating vaccination programs in Laos for the WHO and working on-site during the 1995 Ebola outbreak in the Democratic Republic of the Congo (then Zaire). As part of a hands-on approach, the Agency undertook several studies and surveys to map Covid-19 in the spring of 2020, surveys they used to develop their own forecasts.

Like the team at Imperial College London, the Agency’s in-house group of modelers had access to models developed for influenza that they could have repurposed and used for Covid-19. However, they decided against using them and held off modeling until more data was available. This decision, one senior Agency officer explained, was in part based on an observation that the predictions that had been made for Sweden using available models did not map well against the data coming out of Wuhan and Lombardy:

I believe many took the models for influenza and ran them for Covid-19 and this is what gave them these huge early outbreaks, because that’s what those models were prepared for. But that wasn’t what we were seeing in the data from Wuhan, for example. And when we learned that—after we ran our models with the R-values that were being reported from other countries with active outbreaks and got an outbreak for Sweden that was huge and did not map well with the data we had from Wuhan or from Lombardy—we realized that there was a lot of uncertainty and that we’d better wait until some of the uncertainty had gone away, before we started making our own transmission models, rather than use existing influenza-based models. (Interview E)

However, overcoming uncertainty would not be easy, as there were very little data available on the outbreak in Sweden. The regional healthcare administrations, which were responsible for testing and contact tracing, did not have sufficient stockpiles or resources to carry out large-scale testing programs, which meant that the limited resources at hand were being used selectively. At first, this meant that testing and contact tracing focused on travelers arriving from locations with known Covid-19 outbreaks. Later, as the disease started spreading more broadly, testing capacity was shifted over to hospitals and care facilities. This meant that there was no data available on the epidemiological situation in the broader population. To rectify this, the Agency and the Swedish Armed Forces together launched a series of surveys of the Covid-19 prevalence, first in Stockholm, where the outbreak had its center, and later across the country. The Stockholm survey sampled 707 people between March 27 and April 3 and identified 18 positive tests. The Agency then used a SEIR (susceptible, exposed, infected, and recovered) model to forecast what these results might mean for the near future. The results of these forecasts were published on April 21 in a report estimating that 26% of residents in Stockholm region would be or have been infected with Covid-19 by May 1. It also predicted that the epidemic in the region would peak soon thereafter, at which point the number of infected would decline.^2^

Compared with forecasts developed on the basis of repurposed individual-based models, the Agency’s forecast was based on survey evidence and extrapolation. This version of Covid-19 differed in many ways from those contained in the reports published by the Imperial College team and the team in Sweden. First, the Agency did not include mortality in their forecast, but focused on predicting the future growth, peak, and decline in the number of infected people. Second, by mapping the actual distribution of infected people in a sample (rather than using data from hospital settings or cruise ships as proxies for population data), the Agency’s forecast aimed to address some of the uncertainties regarding the proportion of unknown cases to the number of confirmed cases detected in hospital and care settings. At the same time, the Agency’s forecast had important similarities with the predictions published by others, including by the British and Swedish teams. Like the others, the Agency estimated that Covid-19 would be a rapidly spreading disease with infections doubling at a considerable pace.

The Public Health Agency’s forecast became very influential. Although there were competing visions about what the course of Covid-19 would be—and many experts voiced support for lockdown—the Agency’s voice weighed heavily in the epistemic and policy discussions at the time. However, the Agency was not alone in their views. Another influential prediction, which corroborated the forecast, had been published 4 days prior. In a preprint published on medRxiv, mathematician Tom Britton predicted that infections in the Stockholm region would peak on April 11 and that the number of infected would steadily decrease until 47% of the region’s approximately 2 million inhabitants had been infected, which was estimated to occur by June 15 (Britton, 2020). Together, these projections became highly influential in shaping how Covid-19 was addressed; unlike the individual-based models discussed above, they successfully shaped expectations and supported a particular form of enactment in which epidemiological curves, peak days, herd immunity, and a promise of an eventual return to normal took center stage.

### Herd immunity: Translation and the hope that the pandemic would be over by summer

In the spring of 2020, herd immunity, or the threshold where there is sufficient immunity in a population to make it resistant to the spread of a disease, became a salient theme in debates and media representations of Sweden’s pandemic strategy. This was especially the case as critics began accusing the Agency of actively sacrificing vulnerable people in the pursuit of immunity and an early end to the pandemic (Giritli Nygren & Olofsson, 2021; for an example of such criticism, see Baldwin, 2021). The salience of herd immunity in the debates and media representations of Covid-19 in Sweden began with a Facebook post published on March 13 by former state epidemiologist Annika Linde. In the post, Linde claimed that the Agency aimed to manage the pandemic by isolating and protecting vulnerable people while allowing the disease to spread freely in the healthy population until approximately 60% had been infected and herd immunity was reached. When reporters from *Dagens Nyheter* confronted Anders Tegnell with these claims, he denied that the Agency had any intention to let the disease spread freely, stating:

We absolutely have no ambition to let the disease spread in that manner, we have continuously said that we want a calm and careful spread so that the healthcare sector does not get overwhelmed. Everything we do is completely focused on this. (Lagerwall & Svahn, 2020)

Nevertheless, despite Tegnell’s denial, herd immunity would soon come to dominate media discussions of Covid-19.

Since its rise to notoriety in mid-March, herd immunity became a reoccurring theme at the Agency’s press briefings. While the Agency—together with the National Board of Health and Welfare and the Civil Contingencies Agency—spent the first half of each press briefing providing overviews of the pandemic situation, restating their recommendations, and repeating the importance of protecting the healthcare services by ‘flattening the curve’, the questions raised during the Q&A portion in the second half did not confine themselves to the agencies’ updates and talking points. Instead, journalists asked questions about everything, from how individual residents might best protect themselves or how long football fans would have to wait before they could attend games again, to intricate questions about new research findings and their relevance for pandemic strategies. It was in this context that herd immunity came to assume a key role, being simultaneously a contributing factor to the collective understanding of the pandemic and a promise of a post-pandemic future. One example of this is an exchange between a reporter for BBC News and deputy state epidemiologist Anders Wallensten at the press briefing on April 23:

Reporter: How do you see the potential for herd immunity impacting on the way Stockholmers can live in the coming months in comparison to other countries where people are less likely to be immune following long lockdowns?

Wallensten: It’s a good question. I think it’s too early to say—as I tend to say these days. We don’t know that much about immunity yet. We will know more as more people are tested for antibodies, but also the more time passes and if more accounts of reinfections and etcetera are reported. But as it is, we believe that it has protection at least during the ongoing season. So, of course, many more Stockholmers will be protected than in countries where not so many have been exposed. But I’m not sure that will affect how we live in the short term. Because, as I said, even though maybe 30%, or 23% like this model said, of Stockholmers have been infected in May, it will still mean that the majority have not been infected. (Press briefing April 23, 2020)

At its heart, the discussions of herd immunity in relation to Covid-19 in Sweden relied on the same type of comparison, repurposing, and extrapolation that the predictions and forecasts used. The dearth of data and knowledge about the new disease meant that experts and the public alike used past experiences and assumptions to fill in the gaps in their knowledge and expectations. As with the predictions discussed above, much of the borrowed information came from previous influenza pandemics. This included assumptions on how rapidly Covid-19 would spread through a population, but also assumptions about how many might experience mild symptoms or even be asymptomatic carriers, spreading the disease without knowing they were infected. As in the models, the unknowns and the assumptions used to fill in the gaps contributed to narratives of the epidemiological situation. One example of this is the discussion of how many unidentified cases there were at any given time. If it was true, as some experts argued, that most people would only experience mild symptoms with Covid-19,^3^ then the true number of people infected in the spring of 2020 must have been significantly higher than the number of cases identified through PCR testing in the healthcare and care sectors. Together with the commonplace assumption that Covid-19 would spread very rapidly, the belief that many would be asymptomatic, or experience only mild symptoms fed a popular imagination that promised that if everyone joined together to protect the elderly and other vulnerable groups, it would not be long before the painful present would give way to a brighter post-pandemic future. However, it would eventually become clear that these expectations would go unrealized.

### Collective epistemic work: The Public Health Agency, the media, and the public

By mid-May, when most projections had claimed that Covid-19 infections would be in decline as the epidemic was nearing its end, journalists attending the Agency’s press briefings were raising questions about the Agency’s forecasts. At the May 14 briefing, a reporter from Swedish public broadcaster SR pointed to preliminary results from a Spanish survey indicating that the presence of antibodies to Covid-19 in the Spanish population was far lower than had been expected. Addressing Sara Byfors, the Agency’s representative at that day’s press briefing, the reporter asked whether the Spanish survey gave reason to doubt the Agency’s forecast for Stockholm. Byfors responded saying that it did not and that the Agency was instead looking to expand their survey of Stockholm to a nationwide sample.

Four days later, on May 18, journalists were once again raising questions about the epidemiological situation in Stockholm, this time in relation to preliminary findings from a survey conducted by the Karolinska University Hospital in Stockholm. The university had surveyed its own hospital staff and had found that 15% of the approximately 5,500 PCR tests and 3,200 antibody tests analyzed contained evidence of ongoing or past Covid-19 infections. Armed with these preliminary findings, reporters asked state epidemiologist Tegnell whether the figures contradicted the Agency’s forecasts that 26% of Stockholm’s population would have been infected by May 1. Tegnell responded by pointing out that there is usually a lag between ongoing infection and the development of antibodies, and thus that there was some uncertainty connected to the hospital’s results:

What you’re measuring when you’re looking at antibodies are developments that occurred maybe three weeks ago. That’s how long it takes. So, when you look at antibody prevalence, you need to look at how much has happened until today. We have a doubling time of seven to 10 days, that much we know. Therefore, these 15% would not deviate much from where we believe we are today. But we will consider this in our estimates to see if it fits. (Press briefing May 1, 2020)

Tegnell’s response illustrates how uncertainty affected representations of the pandemic at the press briefings and in the media at the time. In representations of the pandemic’s past, present and future, the lack of stable epidemiological data meant that anyone seeking to pin down and define the pandemic moment was working against a moving target. Because of this, each new publication, preprint, or report created opportunities to revise the state-of-the-art representations of Covid-19. Meanwhile, skeptics would often find ways to use uncertainty to question new findings or argue that it was best to wait and see whether additional information would corroborate them or not. The exchanges between the journalists and Agency officers illustrate the collective nature of the epistemic work involved in understanding, intervening in, and planning for the pandemic’s future development.

As Sweden’s soft lockdown approach depended on voluntary public participation, experts needed to justify their recommendations and the knowledge claims that supported them. The media played an important part in translating and communicating new evidence and recommendations; and as part of this translation work, journalists also drew attention to disagreements and conflicting evidence. Examples of this include instances when reporters asked Tegnell and other Agency officers to comment on the Imperial College model and the UK’s shift to lockdowns (Falkirk, 2020), or to respond to other experts’ criticism of the Agency’s work and recommendations. In other words, while the Public Health Agency had a central role in how Covid-19 was enacted in Sweden, it was not alone in performing this role and was not without challengers.

One important group that regularly challenged the Agency’s knowledge claims and recommendations was a group of virologists, epidemiologists, theologians, journalists, and activists called Science Forum Covid-19 (*Vetenskapsforum Covid-19*). The group aired its criticism on social media, where members gathered in Facebook groups, and in seminars that they broadcasted on Zoom and YouTube. They also raised objections during the Agency’s press briefings. Characteristic of the group’s contributions to the public debate was a hawkish approach to pandemic strategizing, marked by aggressive promotion of new preprints, reports, and research articles, that the group believed might undermine the Agency’s work, or that supported policies such as lockdowns, mandatory face mask rules, and school closures. One prominent member described the group’s work and activism as follows:

The Forum educates the populace in a constructive way, often in contradiction to the eclectic and misleading information from the Agency; produces debate articles; arranges webinars, YouTube programmes and discussions; and shares the state of international knowledge in a understandable way so that people can make their own well-grounded decisions on how to respond. While official leaders have either refused to enter a dialogue or mistrusted the skilled scholars [active in the Forum], journalists and columnists in respectable domestic and international media have monitored their view of the management of the pandemic. (Bergmann, 2022, p. 136)

The clashes between the conflicting viewpoints—that unfolded at the Agency’s press briefings, in news media, and through the spaces organized by Science Forum Covid-19—contradict claims that the Swedish pandemic experience was characterized by a totalitarian consensus culture (e.g. Lindström, 2021; Stilhoff Sörensen, 2023). More importantly it shows that the past, present, and future of the disease remained open, uncertain, and contested, as diverging voices struggled over who was best equipped to define them. Moreover, the multivocality that characterized the public construction of Covid-19 at the time highlights the importance of recognition in who can make claims on the future and influence the present. While the Agency was often able to fend of critics, this did not mean that the Agency was able to dictate the collective understanding of Covid-19; especially as journalists and their audience contributed to the ongoing representations of the pandemic by reinterpreting and re-presenting the Agency’s claims and recommendations. The herd immunity debate illustrates the importance of such reinterpretations. On one hand, herd immunity is an epidemiological term that the epidemiologists at the Agency used to explain how models treat pandemic dynamics, but on the other hand, the term was soon re-interpreted as a strategical commitment and as a prediction for when normality would return. As one Agency officer experienced it:

D: To us—and this probably was a bit nerdy to some degree—but we thought it was important to point out that [immunity] is an important variable when one tries to understand a disease. To help people understand what level of immunity there is in a population and what this might mean in terms of why different interventions produce different effects.

TO: So, this was actually a purely technical question that snowballed into something much bigger?

D: Yes. Like, what will this mean for how long we will have to struggle with this? (Interview D)

## Uncertain but useful: Incorrect predictions and their use

In retrospect, most projections of how the pandemic would develop turned out to be incorrect, adopting Rescher’s (1998) term. For example, the 96,000 deaths that the Swedish team predicted would arise by July 1 did not materialize. Instead, the number of deceased recorded by the end of the week including July 1 was 5,689 (National Board of Health and Welfare, 2023b). However, it was not only the Swedish team’s predictions that turned out incorrect. Most predictions made in the spring of 2020 shared a consensus that Covid-19 would spread rapidly as one large wave of infections and then ebb away by summer. However, while the number of new infections dropped in the summer months, new waves of infections followed. In hindsight, the pandemic in Sweden effectively did not end until February 19, 2022, when the Public Health Agency and the Swedish government lifted the last remaining restrictions and recommendations, with a few exceptions. At that point, 83,978 patients had been hospitalized with Covid-19 and 15,883 people had lost their lives to the disease (National Board of Health and Welfare, 2023a, 2023b). Moreover, despite the enormous suffering that unfolded over the two years of pandemic, the healthcare system managed to weather the storm. The 10- to 40-fold increased demand on intensive care resources and the catastrophic consequences that were predicted to follow (see, e.g. Gardner et al., 2020; Sjödin et al., 2020) never came to be. This was in part due to the efforts of the healthcare providers who, supported by the National Board of Health and Welfare and the Armed Forces, managed to double the number of ICU beds available (Coronakommissionen, 2021). However, it was also because the projections had been incorrect.

When reviewing the reasons why the projections discussed here failed to correctly foretell the pandemic’s trajectory and consequences, some scholars have pointed to the lack of relevant and high quality data (see, e.g. Steinberg et al., 2022), while others have argued that shortcomings both in the models and in the conclusions drawn from their outputs are to blame.^4^ A group of Swedish epidemiologists and modelers, for example, have criticized the Imperial College team for relying on an overly flexible model and for drawing conclusions that went beyond what the data could tell (Soltesz et al., 2020). Nevertheless, while these critical accounts provide insightful lessons for future crises, it is important to realize that, although the projections were incorrect, they were valuable resources at the time. After all, the projections made during the first half of 2020 helped present Covid-19 as a manageable albeit uncertain crisis. As Rhodes and Lancaster (2021, p. 102694) note, models transform the ‘otherwise open and undetermined futures into actionable and plausible trajectories which are situated in relation to the particularities of the material present’. It is therefore relevant to ask not only what went wrong, but also what the futures outlined in models enabled actors to do. For example, the Swedish team’s predictions of devastating consequences unless a lockdown were put in place helped them voice their concerns over whether the current measures and policies would be sufficient. That is, by envisioning Covid-19 as an impending catastrophe and as something that required immediate and decisive action, they successfully transformed the SARS-CoV-2 virus into a political matter (instead of a medical or public health matter), with which they could challenge competing understandings of what Covid-19 was and how it should best be addressed.

In this respect, the projections produced for Covid-19 resemble those made in financial analysis. Financial forecasts, although they can only simulate certainty, nevertheless provide market actors with a sense of predictability in the face of uncertainty. As Leins (2018) argues, financial forecasts act as signposts against which actors can orient themselves when deciding what to do and when. However, while financial forecasts often inform relatively straightforward decisions (e.g. to sell, hold, or buy a particular stock or currency), the decisions facing policymakers, public health experts, and healthcare administrators during the pandemic were far more complex. Moreover, because Covid-19 lacked a history (at least in human populations), the need for sound and accurate predictions was particularly pressing. The strategies and plans available at the start of the pandemic had all been developed with influenza in mind and were increasingly insufficient when used for this new and unknown virus. As one expert modeler explained:

The interest and need to try to understand this thing Covid-19 was much greater than for swine-flu. [With the swine-flu pandemic] you were in part dealing with an influenza virus, so you had a pretty good idea of how it spread. (Interview B)

However, while experts tend to agree that there was a great need to establish an understanding of the new virus, they disagree over what was needed to be made known and for whom it should be made know. The expected mortality, for example, was included in the projections made using variations on the Imperial College team’s individual-based model (e.g. Ferguson et al., 2020; Flaxman et al., 2020; Gardner et al., 2020) but did not feature in the forecasts made by the Agency, nor in the forecasts that the regional healthcare administrations produced. Instead, Agency officers argued against using predicted mortality when creating Covid-19 policy, for two reasons: First, they understood mortality statistics to lack the precision needed to map epidemics. Second, they believed that predicting mortality figures might do more harm than good, as it might provoke unnecessary fear. At the Agency’s press briefing on April 22, Tegnell elaborated on the first objection, arguing that time lags between infection and eventual death, and between the time of death and the registration of a verified death certificate, meant that secure mortality statistics could not be produced quickly enough to be helpful in efforts to mitigate a pandemic. (Press briefing April 22, 2020)

An illustration of the second reason was provided by an Agency officer who explained that the Agency’s projections did not include mortality because they were made with the regional healthcare administrations, the counties, and other governmental agencies in mind:

And we want [the scenarios] to be useful for the regions and counties and other governmental agencies in their planning. So, we have a continuous dialogue with [the organizations] that use the models … about what needs they have for their operations. They have no need for improbable scenarios that depict nightmarish developments. So, [the scenarios we produce each run] are to show probable developments. (Interview E)

Implicit in this discussion is the belief that projections should inform enactments based on information that is relevant and actionable for the organizations responsible for planning and adapting to a changing epidemic landscape. This approach is different from the arguments presented by the modelers making mortality predictions, who used their projections simply to argue for stricter pandemic policies. One example is the preprint published by the Swedish team in which the authors argued that:

Whereas the predictive value of our model should become much clearer over the next two weeks, suppressive measures should be taken more immediately to have a substantial effect, especially for the Stockholm region. The projected mortality from COVID-19 may cause a Swedish all-cause mortality in 2020 exceeding that of 1918, which was the greatest absolute mortality in Sweden in the 20th century. (Gardner et al., 2020, p. 18)

An important difference between the two approaches lies in the audience. While the Agency officer cited above stressed the Agency’s scenarios’ capacity to support decision-making in specialist organizations, the Swedish team sought to mobilize support among politicians and other state actors with the power to influence pandemic strategy and interventions. By addressing different audiences, the two sets of projected futures contributed to the enactment of two distinct versions of the pandemic.

Nevertheless, besides the pragmatic reasons outlined by Agency officers, there is also a moral argument against using mortality predictions in enactments of the pandemic. Some voices claim that modelers, besides producing useful projections, must also take responsibility for the futures that their projections perform. Echoing the Agency officer’s rejection of ‘nightmarish scenarios’, an epidemiologist working for one of the regional healthcare administrations expressed severe doubt, not only about the usefulness of mortality predictions, but their overall moral value:

There were many who made statements about their idea of what should be done and what should not be done; how things would turn out or not, how many would be affected, and how many would die? And you can ask yourself, like, what’s the point of that discussion? Because decision-makers need evidence to make decisions, but some of the evidence that was produced in terms of Covid-19 forecasts during this past year were more of the fear factor type. Like, ‘this is what might happen.’ … Of course you want as few people to die as possible, that’s what we all want. Nobody wants anything other than that. So, to make forecasts of how many that might die just because you can, yeah, I find it hard to understand the purpose of that. That’s not something you can use to make decisions. … Perhaps it’s done with good intent, but the message is not always well received, and instead it might lead to unrest and fear. And the more fearful we are the more we wish to make decisions immediately, if only to show that we are doing something about it, rather than doing things that actually make a difference. (Interview C)

According to this view, predictions that depict a collapsed healthcare system and thousands of dead are epistemologically and morally questionable as they cause unnecessary fear and undermine decision-making. In a period of great uncertainty, when data were scarce and patchy, bold predictions held little sway among adherers of this pragmatic outlook. Instead, the epistemic work carried out within regional and national agencies focused on producing the kind of scenarios and forecasts that they believed would best support their efforts to manage the pandemic within existing frameworks and organizational arrangements. That is, instead of using their predictions to argue for lockdowns or school closures, they used them to support healthcare providers and other actors involved in managing the pandemic’s impacts on the health and wellbeing of patients, care receivers, and others. Moreover, because these predictions had practical use, they had more leverage over the evolving enactments of Covid-19 than others.

As time went by, the Agency found new ways of making its scenarios useful. By the fall, as Sweden faced a second wave of Covid-19 infections, the Agency had established a program to develop near-term scenarios for the demand on healthcare services. While these scenarios primarily targeted the healthcare regions and other government actors, the Agency soon found an alternative audience in the public. As the scenarios received a lot of media attention, the Agency reasoned that they could be used to inspire greater public vigilance and social distancing. As one of its senior officers explained, although the Agency did not develop scenarios directly for the public, it still understood their pedagogical potential:

We produce them for other actors who can use them to plan their operations. However, because the media and the public are interested in learning what might come, we will make use of a pedagogical twist and show also that the developments are not God-given but depend on how we all act. So that’s sort of how they have been used in communication, [to show] that we can influence what will happen. (Interview E)

Interestingly, the officer does not appear to see any resemblances between this ‘pedagogical twist’ and the nightmare scenarios they saw in the predictions made by others. Instead, the scenarios produced by the Agency’s analysts became part of its engagement with the public. Before the pandemic, the Agency’s communication efforts mainly focused on providing recommendations to the healthcare sector, municipal authorities, and local environmental agencies. However, as one senior officer explained (Interview D), during the Covid-19 pandemic, the Agency found itself interacting with a much broader scope of actors, including the public. Through this, the Agency and its scenarios became a central point in the shared epistemic work that unfolded during the pandemic. People tuned in to the press briefings to learn about the latest developments, read about controversies in the media, and were constantly confronted with posters and other information materials reminding them to keep distance and take precaution. While this article has focused on the role of scenarios and predictions in enacting the pandemic, it has only indirectly addressed the public’s participation in this collective epistemic work. Future studies should investigate how members of the public interpreted and contributed to the enactment of Covid-19 in their daily lives and communities.

## Conclusions

During the first half of 2020, when knowledge about Covid-19 was constantly changing, projections of the pandemic’s future played a crucial role in the collective epistemic work through which experts, decision-makers, journalists, and the public enacted the pandemic. By connecting an uncertain present to a potential future, actors constructed meaningful contexts that they could lean on when trying to act in an uncertain world. Projected futures, in other words, helped make the pandemic actionable. In this article, I have sought to outline the background and trajectory of a range of pandemic projections to show how the collective work to define and represent Covid-19 drew upon and created multiple, and at times conflicting, enactments of the pandemic. Through a combination of contextual factors and a continuously evolving knowledge base, some of these enactments grew more influential than others and were able to influence decisions and strategies.

By tracing how representations of Covid-19 changed over time—from the comparisons with past experiences, to the modelers that sought to make the pandemic calculable by repurposing existing models to work for Covid-19, to the survey-based extrapolations produced by the Swedish Public Health Agency—this article highlights the role of different voices and perspectives in the collective epistemic work performed during the pandemic. When viewed through the interactions among various groups of actors, Covid-19 emerged as a multifaceted entity made up of a patchwork of data and assumptions; depending on which futures informed its enactments, the pandemic varied from a catastrophic dystopian scenario to a hopeful promise of an eventual return to normality. Importantly, none of the representations proposed at the time were, so to say, correct. Neither the worst-case scenarios described by some nor the swift return to normal described by others had materialized by the time their predictors had claimed. The broadly held assumptions that Covid-19 would spread rapidly, and that the number of unidentified infections would by far exceed those identified through limited testing programs in place, played an important role in this. These assumptions were central contributors to understandings of Covid-19 at the time and they help explain why so many predictions overestimated the rate of the pandemic’s spread. Importantly, it was also these assumptions that helped transform herd immunity from a technical matter of epidemic dynamics and model specification into a controversy. This was primarily due to misinterpretation, but it also speaks of a longing for a time beyond the pandemic, for immunity, and a return to business as usual.

Regardless of whether projected futures match the actual outcomes, they remain potentially useful. This article echoes what others have already said about the role of projected futures in financial markets and the economy: Projected futures remain useful as long as they contribute to making phenomena navigable. That is, while the projected futures discussed above all turned out to be incorrect, they still allowed actors a degree of navigability in the face of severe uncertainty. However, this also meant that they were only useful and relevant as long as they were seen to be of some practical use. As soon as actors started to question a projection’s practical use, its validity crumbled. This fragility, which is inherent in projected futures, is of central importance to the way that competing enactments came and went in Sweden at the time.

## Notes

1. Other agencies were occasionally present at the press briefings to discuss issues and initiatives that touched upon their areas of expertise. The Agency for Youth and Civil Society, for example, made appearances in relation to a government program providing additional funds to civil society organizations working with vulnerable groups and clients. The Medical Products Agency made repeated appearances in relation to the vaccination campaign that began in late December 2020.

2. The report containing the prediction that 26% of Stockholm residents would have been infected with Covid-19 by May 1 was a revision from an earlier report which estimated that one-third of Stockholmers would have been infected by May 1. The earlier report was withdrawn due to a model specification error that set the number of unidentified cases per identified case too high.

3. Claims that many infected people would be asymptomatic carriers or only suffer mild symptoms of Covid-19 were common in the spring of 2020, although experts were divided on what the consequences of this would be. To some, the large number of asymptomatic carriers was a blessing as it meant that the observable suffering was only a small tip of an otherwise massive iceberg of non-problematic cases. One vocal proponent of this view was the former state epidemiologist, head of research at the ECDC, and current advisor to the Public Health Agency, Johan Giesecke, who argued that only a fraction of those who caught Covid-19 would experience severe symptoms and that 99% would have symptoms so mild that they might not even realize that they had been infected (Larsson, 2020). Other experts disagreed with Giesecke and claimed that the existence of asymptomatic carriers required that Sweden follow the example of other countries and introduce more severe interventions (see, e.g. Einhorn, 2020).

4. For a review of the performance of models predicting the course of the pandemic in Sweden, see Jöud et al. (2021).
